# Restricted antennal movement impacts the tandem running dynamics in a ponerine ant

**DOI:** 10.1186/s12862-024-02267-6

**Published:** 2024-06-13

**Authors:** Snigdha Mukhopadhyay, Subhashis Halder, Eshika Halder, Sumana Annagiri

**Affiliations:** 1 Behaviour and Ecology Lab, Department of Biological Sciences, Indian Institute of Science Education and Research, Kolkata, Mohanpur, 741246 India; 2 Laboratoire d’Ethologie Expérimentale et Comparée, Université Sorbonne Paris Nord, Villetaneuse, 93430 France

**Keywords:** *Diacamma Indicum*, Colony relocation, Recruitment, Tactile sensory impairment

## Abstract

**Background:**

Tandem running is a recruitment method found in some species of ants where one ant follows another ant to reach a destination having maintained a physical contact with its antennae, throughout the journey. It is considered that the exchange of information regarding the destination among the nestmates happened during the process of tandem running. We examined the impact of restricting antennal movement on tandem running by using *Diacamma indicum*, a tandem-running ponerine ant by following 480 tandem runs across 9 treatment colonies and comparing it with 10 control relocating colonies.

**Result:**

Though all the 19 colonies relocated successfully, treatment colonies took significantly longer time to do so. Restricted antennal movement did not influence the ability to become tandem leaders, initiate tandem runs or the work organization significantly. However, antennae-restricted ants performed fewer tandem runs and took significantly longer time. Followers with single or both antennae-restriction performed significantly higher number of interruptions and the alignment between the leader and follower was impacted as antenna-restricted followers subtended a greater angle and walked more to the side of the leader as compared to the control followers.

**Conclusion:**

This study showed unhindered movement of the followers’ antennae is important for tandem-running ants. In the next step, to gain a comprehensive understanding of this recruitment method, it is essential to individually delineate different sensory modalities.

**Supplementary Information:**

The online version contains supplementary material available at 10.1186/s12862-024-02267-6.

## Background

In order to navigate from one location to another animals are known to use a combination of visual, tactile and olfactory cues [1]. Tactile cues are important for several insects for their survival. For example, nocturnal insects like cockroaches are known to use tactile cues from the walls to navigate in the dark [2]. Tactile inputs are used by desert locust (*Schistocerca gregaria*) and Mormon crickets (*Anabrus simplex*) to avoid cannibalism during swarming [3, 4]. Tactile contact from neighbours directs their movement and helps them avoid attacks. Ants use tactile cues for navigation although they rely on visual and olfactory cues as well. Some ant species, such as *Melophorus bagoti, Cataglyphis* sp., and *Myrmecia* sp., use ocelli, to get the directional information from the celestial cues (such as the position of the sun, polarized light from the sky etc.) as back-up mechanism for navigation, when their predominant visual organ i.e., the compound eye was temporarily masked [5–7]. Foragers of the desert ants (*Cataglyphis* sp) use tactile cues associated with their nest entrance during the homebound journey [8]. Army ants (*Neivamyrmex nigrescens*) use tactile cues from objects present on their path along with the chemical cues during colony raids [9].

**Fig. 1:**
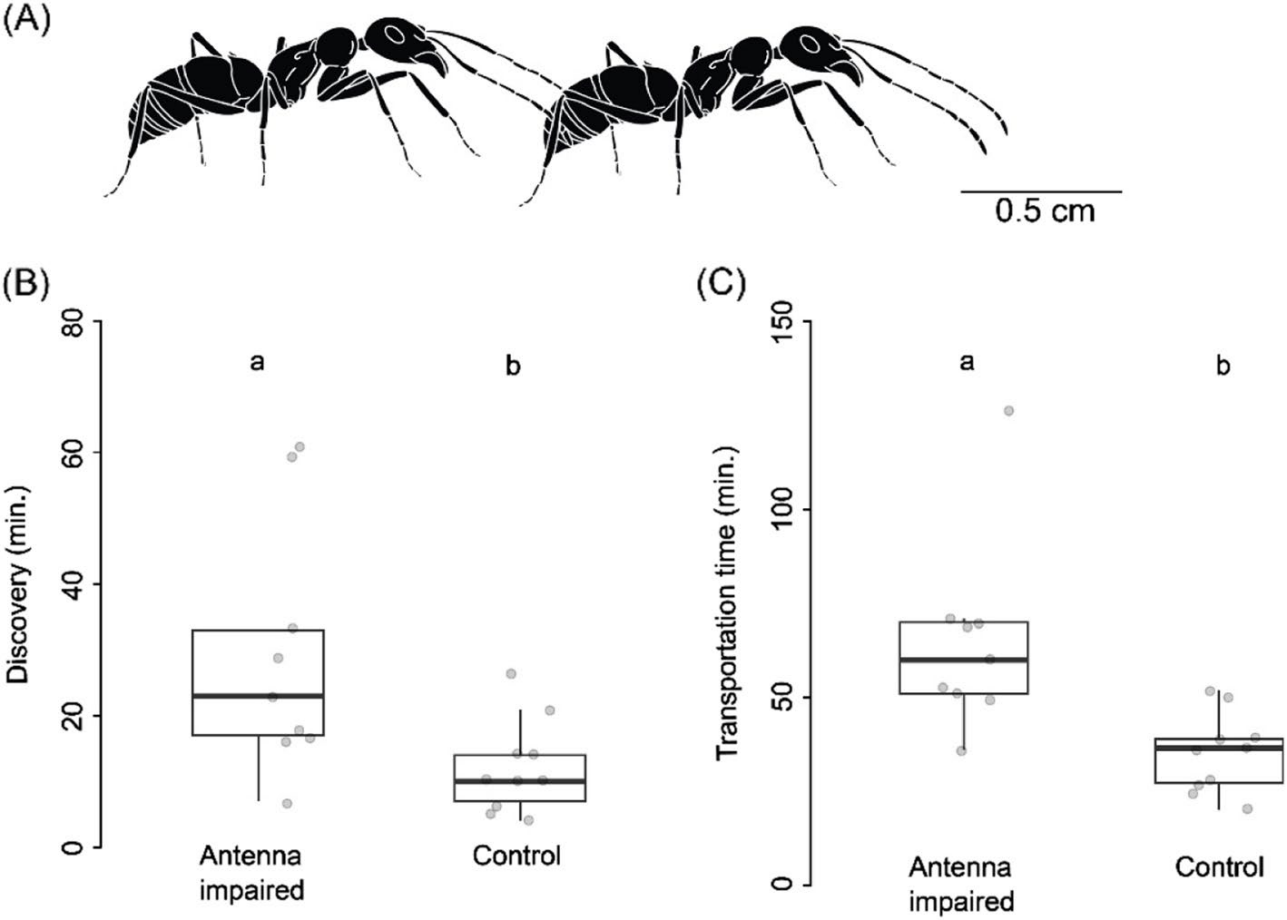
Colony relocation dynamics. Comparison of relocation dynamics between treatment (antennae–impaired, *n* = 9 colonies) and control (*n* = 10 colonies) has been conducted across different categories, discovery (**B**) and transportation time (**C**) have been presented. The different letters above the boxes indicate significant difference between treatment and control relocations (GLM, cut-off value *p* < 0.05). ‘A’ is the schematic illustration of a tandem-pair where the follower is maintaining physical contact with the leader using it’s antennae (Illustration courtesy: Subhashis Halder)

Ants that use tandem running for nestmate recruitment, use tactile inputs to maintain the cohesion between the tandem pairs during (Fig. 1A) the process [10]. Tandem running is a process in which individuals who have information about the destination (tandem leaders) lead their nestmates (followers) to the goal one at a time and throughout the process which does not involve the deposition of a continuous trail pheromone on the substrate, the follower maintains tactile contact mostly with its antennae on the abdomen and perhaps the legs of the leader [11, 12]. However, tandem leaders of *Temnothorax* species are known to release secretions from their poison gland, called “calling pheromone” that helps the process. If the physical contact with the follower is lost, tandem run gets interrupted as the leader will stop and seek its lost follower [13]. Thus, tactile contact between the leader and follower is critical for tandem running. In most insects, tactile cues from the environment are primarily perceived through antennae as many of the mechano-sensory receptors are concentrated in the antennae and sparsely scattered on their legs and other body parts as well [14]. Although some researchers consider tandem running as a relatively primitive mode of recruitment in ants [13], it is a method of recruitment used by several species to recruit nestmates in the context of foraging, colony relocation and slave raid [15]. Mostly species with smaller colony size are known to use this mode of recruitment, as they are unlikely to have enough colony members to generate the concentration gradient of pheromones required to maintain trails over time. Tandem running recruitment can also provide other advantages, unlike chemical trails the paths used in tandem running cannot be tracked by the predators [16]. Moreover, tandem running can be used across diverse terrains even when it is constantly raining.

This study was conducted to unveil the importance of antennal movement during tandem running in the context of colony relocation in *Diacamma indicum*. This 1 cm long, black, ponerine ant is found in the India, Bangladesh, Sri Lanka, and possibly Japan. Tandem running is used by these ants exclusively for colony relocation [17]. Previous studies on vision impaired *D. indicum* showed that these ants relied on thigmotactic cues to conduct tandem running and successfully relocated their nestmates in the absence of visual inputs [18]. During relocation in this species, while all adult females are tandem run, the brood items and the males are either carried by the adult female scout or by the followers by their mandible to the new nest [19]. Moreover, colony relocation causes exposure of the whole colony along with the reproductive individual and brood to the harsh biotic and abiotic environmental factors. Colony fitness is directly dependent on the efficiency of relocations, as delay in the process makes the colony vulnerable to predators, parasites and thieves [20]. Hence, the relocation process is expected to be well optimized in terms of navigating to the new nest.In order to examine the importance of antennal movement during tandem running we specifically asked four questions. First, we examined if these ants are able to perform tandem running with restricted antennal movement. Next, we examined the effect of restricted antennal movement on the overall dynamics of the relocation process. We examined if antennal impairment affects the recruitment and performance of tandem leaders. Finally, we inspected how restricted antennal movement affects the execution of tandem run. All experiments were carried out inside the laboratory together with control experiments in which ants without any restriction on their antennae, performed the same task. To understand these features, we conducted analysis at both the colony and individual level.

## Result

### Relocation dynamics

All the 19 colonies relocated successfully across the control and treatment relocations while maintaining colony cohesion, as no nestmate was left behind or lost. After analyzing the parameters, it was found that antennae impairment impacted the discovery time and transportation time significantly in treatment relocation compared to the control relocation. The discovery time in control (12 ± 7.04 min) (Mean ± SD) was approximately 1.5 times faster than the treatment relocation (29.22 ± 19.02 min), (GLM, t = 2.21, *p* = 0.04; see Table S1; Fig. 1A); and transportation time in control (35.2 ± 10.59 min) was about 2 times faster than that of in treatment relocation (65 ± 25.64 min) (GLM, t = 2.75, *p* = 0.01, see Table S2; Fig. 1B). The percentage of individuals that became explorers and leaders, were not statistically different in treatment and control group (Table S3).

Work allocation was analyzed by examining the individuals that became leaders and how they distributed the associated jobs into different categories. The percentage of leaders that emerged from no antennae impaired category was statistically not different than that from the other two categories of individuals. Average percentage of leaders with both antennae-impaired, single antenna-impaired and no antenna-impaired were 20.38 ± 11.08, 26.21 ± 10.14 and 53.41 ± 19.21 respectively (Friedman Test, χ2 = 9.58, df = 2, *p* < 0.01; Post hoc. Wilcoxon paired sample test with Bonferroni’s correction, both vs. single, *p* = 0.17; both vs. none, *p* = 0.02; single vs. none, *p* = 0.03 (following Bonferroni’s correction for a significant difference, p should be lower than 0.02)). The percentage of tandem run performed by both antennae-impaired leaders (14.51 ± 10.64) was significantly lower than the leaders with no antennae impairment (58.27 ± 27.12), while single antenna impaired leaders (27.21 ± 20.70) did an intermediate level of transportation. (Friedman Test, χ2 = 9.58, df = 2, *p* = 0.02; Post hoc. Wilcoxon paired sample test with Bonferroni’s correction, both vs. single, *p* = 0.09; both vs. none, *p* = 0.01; single vs. none, *p* = 0.09 (following Bonferroni’s correction for a significant difference, p should be lower than 0.02)).

**Fig. 2:**
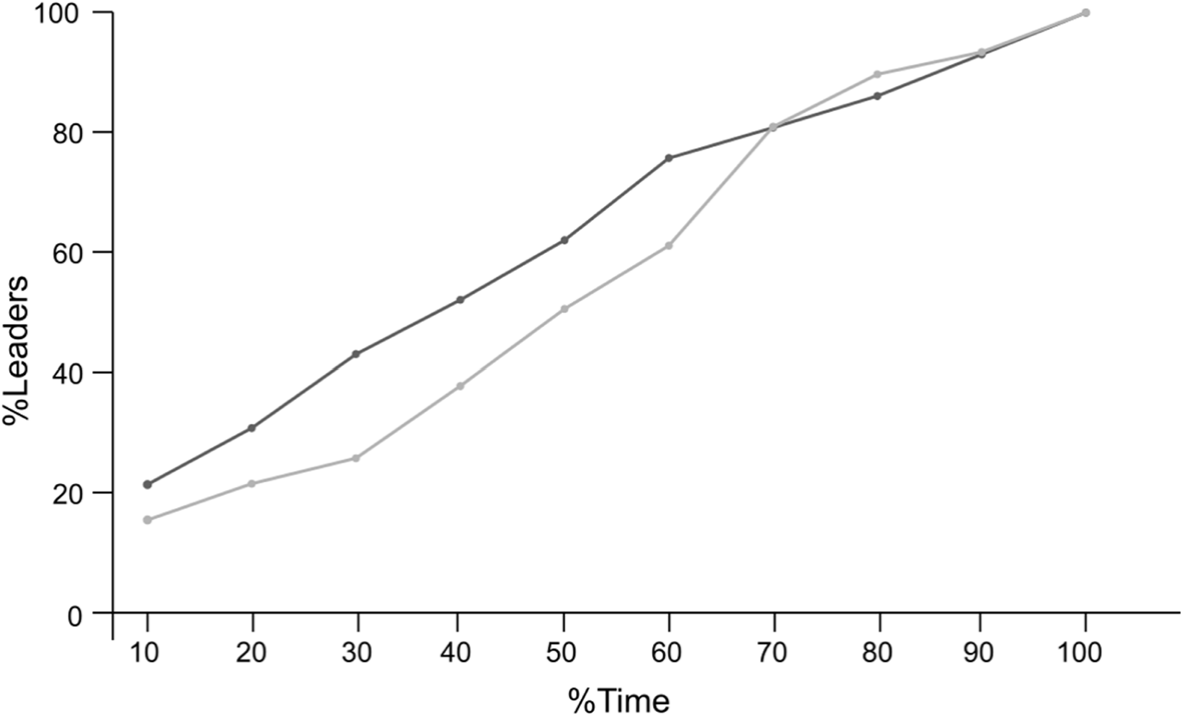
Leader recruitment dynamics. Comparison of tandem-leaders recruitment over the total transportation time has been represented using a line graph across antennae-impaired (treatment, *n*= 9) and control (*n*= 10) relocation. The x-axis represents the percentage relocation time divided into bins of 10%, and the axis spans from the start of the first transport (0%) to the last transport to the new nest (100%). The y-axis represents the cumulative percentage of recruited leaders across treatment (black line with dot) and control (grey line with dot) relocation (GLS, cut-off value *p* < 0.05)

Relocation progress was analyzed further as the transportation time was significantly higher in the treatment. The reason for this delay was investigated by examining two parameters here. The manner in which the tandem runs progressed over time was examined by considering the progress of leader recruitment (GLS, t = -1.52, *p* = 0.12, see Table S4; Fig. 2) and progress of transports (GLS, t = -0.09, *p* = 0.34; see Table S5) over time in treatment. It was found that neither of these parameters were significantly different. The delayed discovery and transportation can be a result of movement hindrance due to the antennal impairment. The results showed that total distance travelled by the individuals was not influenced by the anetennal impairment, but the furthest distance travelled by the no-antennae impaired ants was significantly higher than the both-antennae impaired ants (see ‘Mobility assay’ in Supplementary material SE1).

### Efficiency of individual tandem run

The delayed transportation time can be a result of lower efficiency of individual tandem runs by antennae-impaired leaders as well as the followers. In order to examine this, we further analyzed the three parameters at the level of individuals, such as - the time taken for initiating a tandem run, and the number of interruptions during a given tandem run and the time taken to complete a single tandem run. Across 9 colonies in antennae-impaired relocations, 546 successful tandem runs were considered for the following analysis. We conducted this analysis by examining the leaders and followers separately.

### Initiation time

In order to understand the impact of leaders’ impaired antennae, we investigated the outcome of tandem runs performed with followers who had both their antennae unimpaired. Average time for initiating a tandem run by both, single and no antennae-impaired leaders was 5.89 ± 1.96 s, 7.12 ± 3.68 s and 7 ± 4.5 s respectively (Kruskal-Wallis test, χ^2^ = 0.12, df = 2, *p* = 0.94). To understand the impact of impaired antennae on followers, we investigated the outcome of tandem runs performed with leaders who had both antennae unimpaired. On average, 8.5 ± 5.5 s, 6.27 ± 3.57 s and 6.79 ± 4.33 s were taken to initiate tandem runs with both, single and no antennae-impaired followers (Kruskal-Wallis test, χ^2^ = 2.49, df = 2, *p* = 0.29). This result showed that the antennal-impairment in the leaders as well as in the follwers did not affect the time taken to initiate individual tandem run.

### Number of interruptions

**Fig. 3:**
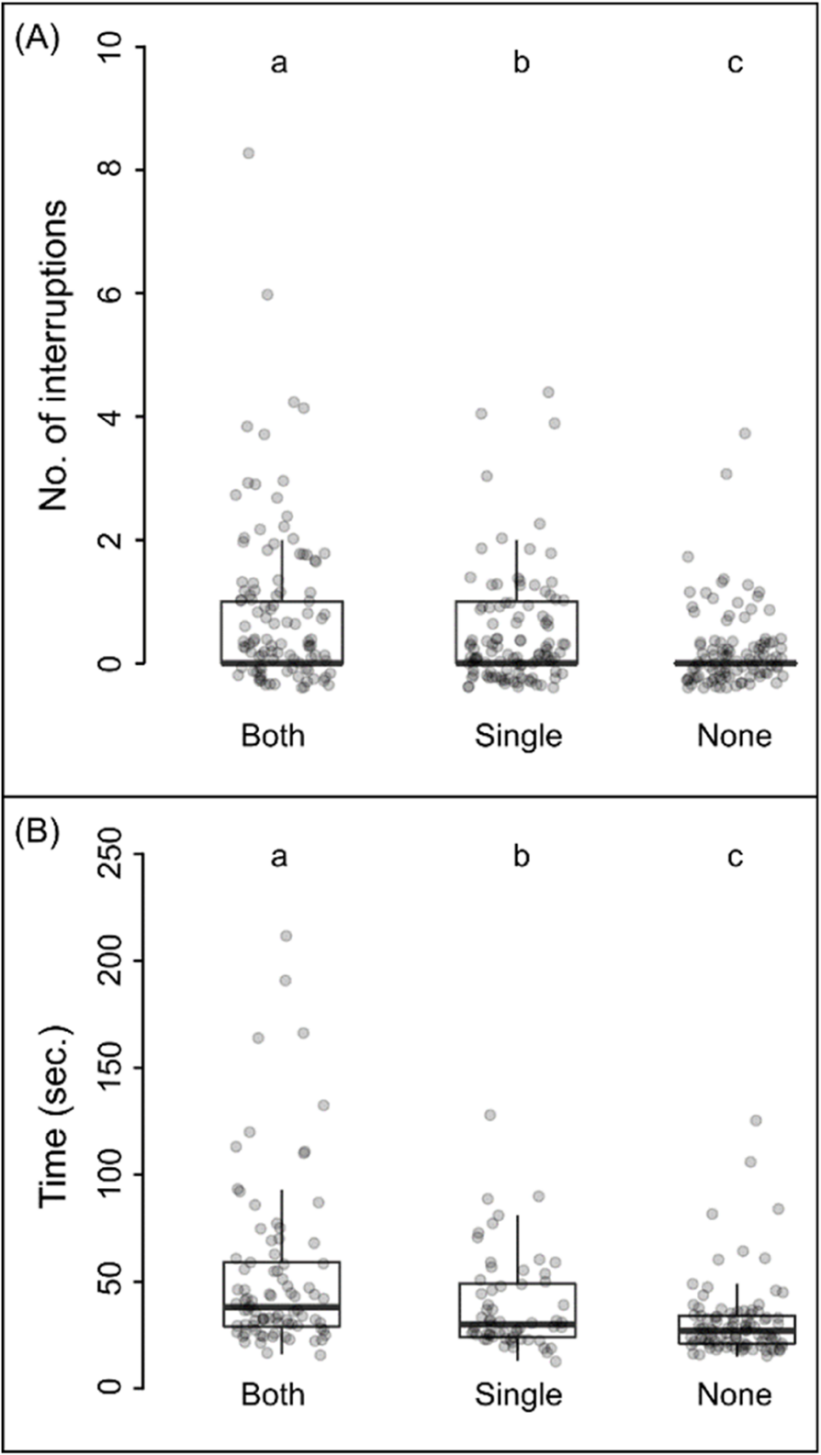
Tandem running efficiency. Comparison of efficiency of individual tandem runs where followers have both (*n* = 104), single (*n* = 107) or no-antennae (*n* = 101) impaired and leaders have no antennae impairment, is represented for interruptions that occurred during a tandem run (**A**), and time taken to complete a tandem run (**B**). The different letters above the boxes indicate significant difference between treatment and control relocations GLM, cut off value *p* < 0.05)

Average number of interruptions that occurred during a tandem run was influenced by the antennal impairment condition of the tandem pair (GLM, z = 3.75, *p* < 0.001; Fig. 3 A, see Table S6). Average number of interruption was 1.76 ± 1.85 when a both antennae-impaired leader leading a both antennae-impaired follower to the new nest which is significantly lower compared to a both antennae-impaired leader leading a no antennae-impaired follower (0.11 ± 0.32) (Pairwise comparison, *p* < 0.001; for detailed statistics see Table S6). Details of average number of interruptions that occurred during a tandem run across all the 9 categories of tandem pairs were given in Table 1.

**Table 1:** Details of average number of interruptions occurred and average time taken to complete a tandem run is given below

Tandem pair (leader-follower antennal impairment condition)	No. of interruptions	Time
Both-Both (bb)	1.76 ± 1.85	86.06 ± 51.29s
Both-Single (bs)	0.76 ± 0.90	48.17 ± 21.72s
Both-None (bn)	0.11 ± 0.32	44 ± 61.86s
Single-Both (sb)	1.28 ± 1.91	56.69 ± 57.77s
Single-None (sn)	0.15 ± 0.36	27.84 ± 10.15s
Single-Single (ss)	0.37 ± 0.70	34.82 ± 23.85s
None-Both (nb)	0.95 ± 1.39	52.66 ± 38.74s
None-Single (ns)	0.49 ± 0.87	39.37 ± 21.77s
None-None (nn)	0.25 ± 0.63	31.82 ± 23.84s

### Time

Average time taken to complete a tandem run was influenced by the antennal impairment condition of the followers (GLM, t = 45.50, *p* < 0.001; Fig. 3 A, see Table S7). Average time taken to complete a tandem run was 86.06 ± 51.29s when a both antennae-impaired leader leading a both antennae-impaired follower to the new nest which is significantly lower compared to the same of a both antennae-impaired leader leading a no antennae-impaired follower (44 ± 61.86s) (Pairwise comparison, *p* < 0.001; for detailed statistics see Table S7). Details of average time taken to complete a tandem run across all the categories of tandem pairs were given in Table 1.

### Tandem pair alignment

**Fig. 4:**
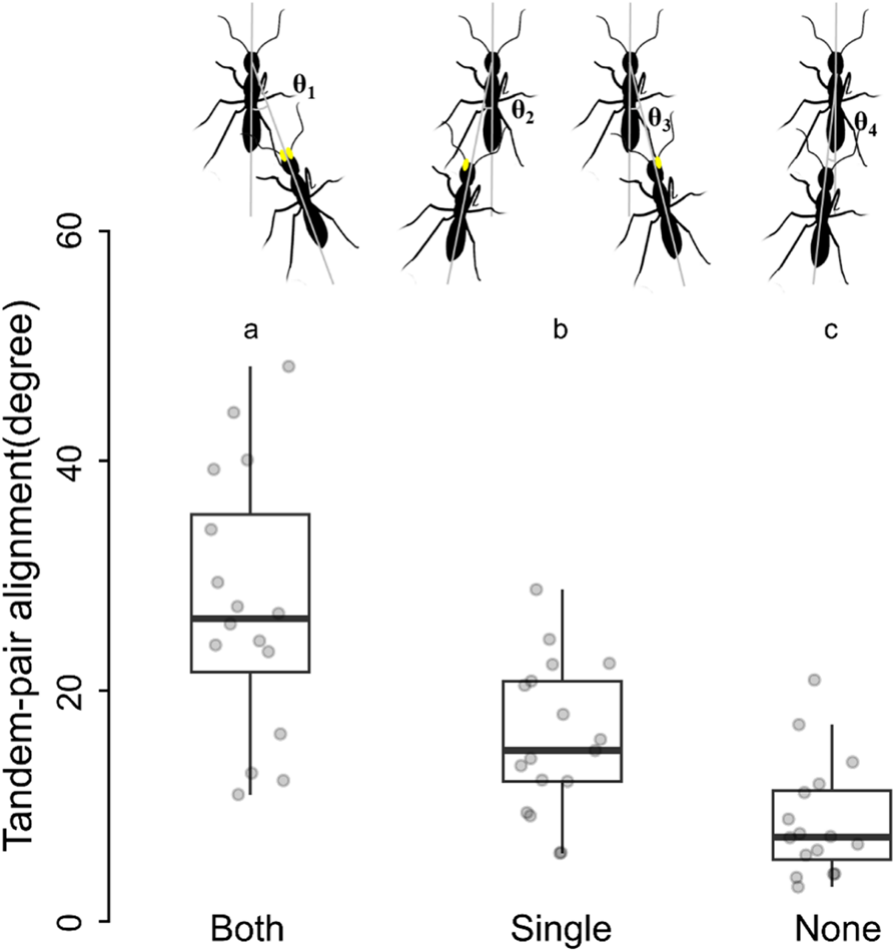
Alignment of leader-follower during tandem running. The schematic of tandem pair alignment has been shown in the upper panel where a solid ‘yellow circle’ on the followers indicates the antennal restriction, and the light grey lines on the ants are considered as the body axis. The angle between the follower’s body-axis with the leader’s body axis is marked as θ_1_ in the case of both antennae-impaired, θ_2_ and θ_3_ in left and right single antennae-impaired and θ_4_ in no manipulation. Comparison of alignment angle of individual tandem pairs where followers have both- (*n* = 15), single- (*n* = 16) or no-antennae (*n* = 15) impairment. The different letters above the boxes indicate significant difference between treatment and control relocations (GLM, cut off value *p* < 0.05)

In order to understand the higher number of interruptions occurring due to the impairment in followers’ antennae, we zoomed into the position of the followers while they followed the leader to examine the distance between the abdomen of the leaders and head of the follower and their alignment were compared across all 3 categories. The average distance between leader and follower of a tandem pair was statistically comparable across all 3 categories of followers. For a given tandem pair, the average distance between the abdomen of leaders and the head of follower with both, single and no antennae restricted follower was 0.27 ± 0.13 cm, 0.32 ± 0.09 cm and 0.29 ± 0.11 cm (Kruskal-Wallis test, χ2 = 2.06, df = 2, *p* = 0.36). The average alignment angle of a leader-follower pair (*n* = 48) alignment with no antennae-impaired followers was significantly lower than cases in which both and single antennae-impaired followers. Average angle of leader-follower pair alignment with both, single and no antennae-impaired follower was 27.44 ± 11.36°, 15.91 ± 6.59° and 8.73 ± 5.07° (GLM, t = -4.71, *p* < 0.001; Fig. 4, lower panel, see Table S8).

To further investigate the impact of the tandem pair alignment angle on the interruption of tandem runs, Pearson’s correlation analysis was done between these parameters across these 48 individual tandem runs, and the result showed no. of interruptions was positively correlated with the tandempair alignment angle (*r* (47) = 0.72, *p* < 0.001).

## Discussion

In this study, we wanted to investigate the impact of restricted antennal movement on tandem recruitment. To the best of our knowledge, this is the first study to focus on navigation during colony relocation in a tandem-running ant species involving the restriction of antennal movement.

This study showed that colonies even with restricted antennal movement performed tandem runs and successfully relocated to their new nest at the cost of additional time with reduced tandem running efficiency. Transportation time was approximately 2 times longer as compared to control colonies in which ants did not have any restriction on their antennal movement. A recent study on visually impaired *D. indicum* showed that these ants relied on tactile cues from the walls of the arena as they walked along longer routes to the new nest, further in the absence of enroute tactile cues, vision impaired colonies were unable to relocate. This indicated that tactile cues acted as a backup when visual cues were missing [18]. When the opposite treatment was performed in the current set of experiments, i.e., when antennae were restricted and eyes were unimpaired, ants took significantly longer time to perform tandem run but qualitatively they did not show any particular preference to walk along the edges of the arena nor did they avoid the edges, which indicates that the ants with functional eyes does not have a preference to walk along the edges.

In the next step, we tried to find the reasons that caused prolonged transportation time in case of colonies with restricted antennal movement. On examining individual tandem runs, we found that percentage of successful tandem runs was not significantly different from the control relocation, although a high number of interrupted tandem runs occurred in treatment relocation depending on the condition of the followers’ antennae. This indicates that tandem leaders and followers even with impaired antennae can search for each other and reinitiated tandem run when they got interrupted. At the level of individual tandem runs, we found that tandem pairs with followers having restricted antennal movement, either both or single antenna completed tandem runs with lower efficiency in terms of time taken to complete and the number of interruptions during the tandem run. During tandem running, followers maintain physical contact with leaders throughout the journey [12]. Perhaps, this physical contact was disrupted when the follower has impaired antennae, however the impairment done in the current experiment was not so severe as to hamper their ability to become followers altogether. Either by adjusting the distance between the leader and the angle subtended between the leader and follower, they are able to progress with tandem running. We did not find a significant difference in the distance between leaders and followers as compared to control, so this was not impacted, but the alignment between leader and follower was impacted by the antennae impairment of the followers. Followers with impaired antennae probably faced difficulties in maintaining physical contact with the leaders, thus they shifted themselves to one side of the leader causing the angular distortion between leader and followers. Perhaps followers compensate for restricted antennae movement by using other appendages like mandibles or legs to stay in touch with the leaders. Additional experiments with more severe antennal impairment or absence of antenna altogether with detailed analysis of the tandem runs itself, are required to further understand how these ants compensate for restricted movement of their antennae.

Although, the antennal impairment did not influence the capability of the individuals to become leaders during transportation, it had a significant impact on the amount of work done by the individual leaders. Leaders with no-antennae impaired performed a significantly higher number of transportation than the leaders with both antennae-impaired. Surprisingly, the progress of leaders’ recruitment and transport of colony members over time was not significantly different across treatment and control relocations, which indicates that colonies maintained the relative pace of transportation over time even when their antennae were impaired.

Tandem running is considered as the first example of teaching in non-human species [15], where the tandem leaders teach the followers to navigate to their destination by transferring the information, which in turn changes the behaviour of the followers allowing them to become leader themselves. In *T. albipennies*, transferring the information regarding their new nest and the route to fellow nestmates depends on visual cues [21] while visually-impaired or blind *D. indicum* leaders were able to transfer the information to their blind followers, in the presence of thigmotactic cues [18]. Results from this study showed that the restricted movement of the antennae of the colony members did not influence the ability to become a tandem leader, although the work capacity was significantly affected. This allows us to conclude that, restricted antennal movement do not hamper leaders from teaching and followers from learning the route.

## Conclusion

This study found that, despite having restricted antennal movement, without any major impairment of the sensory inputs on the antenna in *D. indicum*, they successfully performed the goal-oriented task of colony relocation while maintaining the colony cohesion, but at the additional cost of transportation time. Tandem leaders compensated for followers’ impairment by performing slower tandem runs and resuming interrupted tandem runs which led to an overall increase in transportation time. Ants with restricted antennal movement were able to recruit other members to the task and the work organization mostly remained unaltered as the control relocation. Exploration of leaders and followers’ performance with targeted impairment of the inputs from the antennae, along with experiments with multimodal sensory impairment can be used to further understand how the information is passed on to the followers from the leaders and how tandem pairs maintain the cohesion through the process.

## Materials and methods

Nineteen *Diacamma indicum* colonies were collected from Nadia, West Bengal, from May 2018 to August 2019 using the nest flooding method [19] and were brought to the laboratory and maintained following the standard protocol with *ad libitum* food and water [22] for experimental purpose. Each colony was examined under microscope to discover the single reproductive individual called gamergate, and only colonies with gamergate were used for the experiments. All adult females of the colony were marked on one or more of their body parts (1st and 2nd thoracic segments and abdomen) with non-toxic enamel paint (Testors, Rockford, IL, USA) to give a unique identification. Each colony was used for a single relocation.

### Experimental set-ups

A 60 cm X 90 cm arena, which had a base consisting of a mixture of soil and sand, was used for all the relocation experiments. In this arena, the old nest (the nest containing the colony) and the new nest (an empty nest, that was identical to the old nest) were placed at the diagonally opposite corners. The experimental set-up and initiation of the relocation process were identical to the procedure as described in Mukhopadhyay and Annagiri, 2021 [21] and was uniformly applied across all the replicates of both treatment and control experiments. The entire relocation process was recorded by using video recorders (Sony Handycam, model: HDR CX200).

The aim of this experiment was to examine the impact of restricted antennal movement on the efficiency of the relocation process and precisely that of the tandem leaders and followers. To address this, we have conducted two sets of relocation experiments as described below.

### Antennae impaired relocation (treatment relocation)

**Fig. 5:**
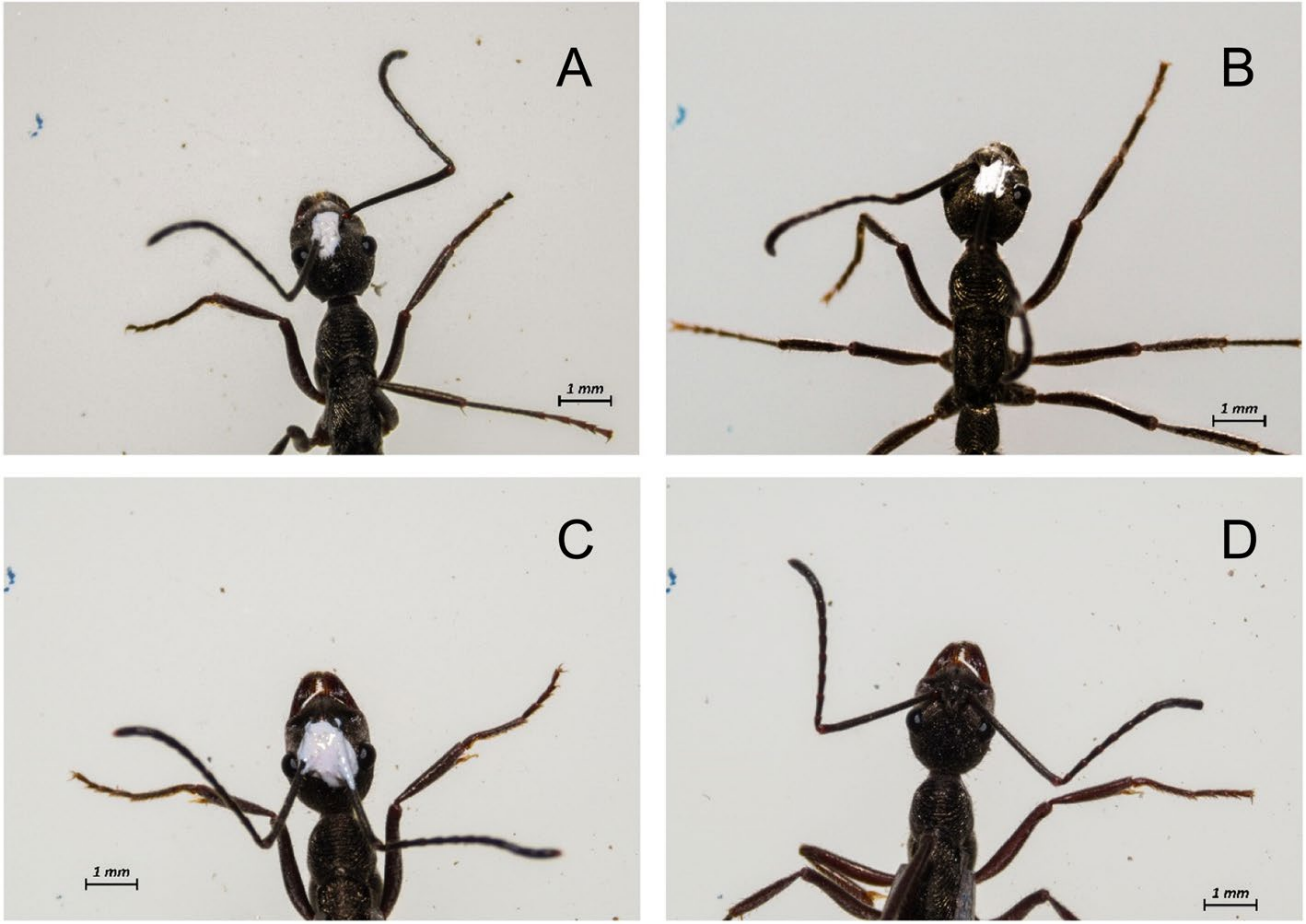
Pictures depicting antennae impaired and normal *Diacamma indicum* workers. **A** shows a worker with an impaired left antenna, **B** shows a worker with a right antenna restricted, and **C** shows a worker with both antennae that have been restricted. **D** shows an unrestricted ant (control) worker with both the left and right antenna intact. The antennae were fixed at their base using non-toxic enamel paint to restrict the antennal movement. Note that the ants were positioned with their head facing upwards and orientation of the antennae was considered in this position

Nine colonies having 75.77 ± 28.45 adults, were used in this set of relocations which was also referred as the treatment relocation. Before conducting the relocation, all the adult females of the colony were randomly assigned into three categories. Within a colony, 1/3rd of the members received both antennae restriction, 1/3rd of the colony members received single antennae restriction (50% right and 50% left antenna impaired) and the remaining 1/3rd part of the colony received no manipulation of their antennae. The process of antennal impairment was done 10 to 12 h before the start of the relocation. The marked ants were separated according to the category into glass vials individually and were given cold shock for 15 min by placing them in an ice bucket. This made the ant temporarily unresponsive, which helped us in impairing their antenna. All the individuals of the colony went through the same cold shock irrespective of being in both-antennae, single-antennae or no-antennae categories. (A small drop of non-toxic enamel paint was applied at the base of the antenna of the ant to restrict antennal movement with a dissection pin. More specifically, the scape was glued to the head capsule. This treatment allowed the ants to move other segments or the flagellum of their antenna, but the reach of the antenna was compromised. However, other functions of the antenna were not hindered, and they are likely to respond to thigmotactic and olfactory cues in a similar manner as unmanipulated ants. Thus, these ants had a lower degree of antennal movement but were “normal” in all other respects. The various treatments can be seen in Fig. 5. All the members of the colony were examined 2 h prior to the relocation experiment, to confirm that they had not groomed off the paint from the antennae, and if they had groomed off the paint, their antennal base was again fixed with paint. After 48 h, we found that almost all ants had removed the paint from the base of their antenna and this treatment thus only had a temporary effect. The same paint was used to mark different parts of the ant’s body in order to achieve individual identification. Thus, the ants who did not receive any paint on their antennae acted as control for cold exposure and as sham control for the application of paint on the body of ants [6].

### Control relocation

Another ten colonies with 94 ± 38.67 adult female, were used in control relocation in which no colony members neither received any exposure to cold nor did they receive any impairment to their antennae, except having paints on their body part for individual identification. All the conditions of the relocation experiments, starting from the initiation of the relocation to data collection, were kept identical across the treatment and control sets. The colony sizes in antennae-impaired and control relocation were not statistically different (Mann Whitney U test, U = 57, N1 = 9, N2 = 10, *p* = 0.35), thus any difference we see in the behaviour between these two sets will not be attributable to differences in the colony size.

### Behavioural observation

Behavioural observation was conducted by using real-time data collection during the experiment and video recorders. Data was decoded from the recordings into datasheets and digitalized by entering them in Microsoft excel 2013 (Windows 10). To analyze the parameters at the level of colony, information regarding the time at which the colony was placed inside the arena, the time when 1st individual of the colony discovered the new nest and its identity, the time when 1st tandem pair reached the new nest (the start of transport), last tandem pair reached the new nest (end of transport), identity of the individuals discovered the new nest before starting the transportation and identity of the leader-follower pair in all tandem runs were recorded across all relocations. Based on the inputs, parameters like discovery time, transportation time, percentage of the colony who became explorers, percentage of the colony who became leaders, and percentage of successful tandem runs, were calculated (Table S9) [21, 26]. To analyze the colony level parameters like discovery time and transportation time between antennae-impaired and control relocation, a generalized linear model (GLM) was used. Antennal condition was used as predictor to analyze the “discovery time” as the response variable. On the other hand, colony size, discovery time and antennal condition were used as predictors to analyze the “transportation time” as response variable. For analyzing remaining parameters, such as percentage of the colony became explorers, and leaders, and percentage of successful tandem runs, two-tailed nonparametric Mann Whitney U test was used.

The time taken for every tandem run was also recorded. If antenna impairment did not play a major role in initiating tandem runs, we expected comparable number of leaders to emerge from these three categories in treatment colonies. The percentage of ants that became leaders emerged from each category was calculated for each colony. Also, number of tandem runs performed by the leaders of 3 different categories were recorded and from this data, percentage of tandem runs done by 3 categories of leaders were also calculated. To compare these parameters across treatment and control relocation, two-tailed nonparametric Friedman test was used, and post hoc. Wilcoxon rank-sum test was used to compare within categories.

In general, followers maintain continuous contact throughout the tandem run. A leader-follower pair that was initiated at the old nest and ended to the new nest, is considered as successful tandem runs. If the initial leader-follower pair got separated from each other and lost physical contact with each other (for more than 3 s and less than 15 s) but resumed their tandem run after this separation, the tandem run was designated as interrupted tandem run. The number of such interruptions was recorded, and these interrupted tandem runs were also considered successful tandem runs, as the initial tandem pair that started from the old nest, reached the new nest. The percentage of successful tandem runs were calculated based on these data. All the followers were also categorized into three conditions, both antennae or single antenna or no impairment and percentage of interruptions occurring with followers in each of these categories was calculated.

Next, in order to examine the influence of the antennae impairment on the overall work distribution of the colony, two parameters, e.g., percentage of leader participation and percentage of transportation over time were analyzed for both treatment and control relocations. If antennae restriction impacts leader recruitment or transportation dynamics the progression in the treatment graph would be different as compared to controls. The number of unique leaders and number of tandem runs that occurred at every 10%-time interval of the total transportation time was noted and analyzed [21]. To compare the work distribution between antennae-impaired and control relocation, a generalized least square (GLS) model was used for both the parameters. Interaction of antennal condition and time progress was used as influencing factor for the response variables (leader recruitment and transportation progress).

The following parameters were analyzed to examine the effect of antennal impairment on the efficiency of the individual tandem runs (*n* = 546). Time taken for each tandem run and the number of interruptions that occurred was calculated and categorized according to the condition of the antennae for the leader and follower in a tandem pair. The calling or invitation time to initiate a tandem run were recorded and calculated for individual leader-follower pair. Tandem running invitation is given by tandem leaders who were aware of the new nest’s location. This is typically a jerky movement performed by the tandem leaders towards potential followers, which includes pulling of antennae, leg and body of the potential follower [10]. In *D. indicum* leaders make four to five calls towards three individuals and take 16 s on average to find a follower [23]. We hypothesized that antennal condition would play a role in this process. Specifically, leaders with both their antennae impaired would probably not be able to make the appropriate antennal movements and would be expected to have problems with inviting followers. In order to address this question, we recorded the time taken to invite followers for tandem running. We randomly sampled 25% of total tandem runs of each colony (*N* = 115 tandem runs across all 9 colonies) to examine the invitation time. To analyze these parameters across all 9 categories of tandem pairs, two generalized linear models (GLM) were created with number of interruptions and time taken to complete a tandem run as dependent factors and type of tandem-pairs as fixed factor.

Due to the impairment of the antennae, we observed that the alignment of the follower with the leader during the tandem run was qualitatively different as compared to the control followers. In order to investigate this further, we measured the angle subtended between the follower and the leader while they were tandem running (termed as tandem pair angle). Tandem pair angles were measured by taking screenshot of the leader-follower pairs during tandem running and then with the help of ImageJ software, we drew two straight lines along the body axis of the leader and the follower. The intersection of these two extended body axes was considered as the angle of the tandem pair. For this analysis we randomly selected 48 tandem runs (10% of total tandem runs recorded) across all the 9 treatment colonies and took 4 screen shots along their journey from the old to the new nest. The follower alignment angle across the three categories, both antennae (*N* = 15), single antenna (*N* = 17) and control (*N* = 16) ants were compared (Fig. 4 upper pannel). From these same images the distance between the abdomen of the leader and the follower’s head was also measured using ImageJ software, with the ant itself acting as the scale. To analyze these parameters across all 9 categories of tandem pairs, a generalized linear model (GLM) was created with angular aligmnet of a tandem-pair as dependent factors and type of followers as fixed factor. A value of *p* < 0.05 was considered as the cut off value for statistical significance for all the comparisons. Mean and standard deviation values are presented unless mentioned otherwise.

### Supplementary Information

Supplementary Material 1.

## Data Availability

All the experimental data are available only on reasonable request.
