# Supplementary material for: Restricted antennal movement impacts the tandem running dynamics in a ponerine ant
Source: BMC Ecol Evol. 2024 Jun 13;24:82. doi: 10.1186/s12862-024-02267-6 (PMC11177493; doi:10.1186/s12862-024-02267-6)
Supplement: Supplementary file 1 — Supplementary Material 1. [file 12862_2024_2267_MOESM1_ESM.docx]

**Restricted antennal movement impacts the tandem running dynamics in a Ponerine ant**

Snigdha Mukhopadhyay^1,2^, Subhashis Halder^1^, Eshika Halder^1^ and Sumana Annagiri^1*^

^1^ Behaviour and Ecology Lab, Department of Biological Sciences, Indian Institute of Science Education and Research, Kolkata, Mohanpur-741246, India.

^2^Laboratoire d'Ethologie Expérimentale et Comparée, Université Sorbonne Paris Nord, Villetaneuse-93430, France

Phone: 91-33-66340000 ext 1203

^*^Corresponding author’s email: [sumana@iiserkol.ac.in](mailto:sumana@iiserkol.ac.in)

**Supplementary information**

**Table S1: Details of GLM analysis to investigate the factors impacting the discovery in control and antennae-impaired relocation experiments**.

Model <- glm (Discovery ~ Ants’ antennae condition, data= discovery, family= gaussian (link = log))

| **Coefficients** | **Estimate** | **Std. Error** | **t value** | **p value** |
| --- | --- | --- | --- | --- |
| Intercept | 2.48 | 0.37 | 6.73 | < 0.01 |
| Ant_antennae_impaired | 0.89 | 0.40 | 2.21 | 0.04 |

**Table S2: Details of GLM analysis to investigate the factors impacting the transportation time in control and antennae-impaired relocation experiments**.

Model <- glm (Transportation_time ~ Colony size + Discovery + Ants’ antennae condition, data= transportation_time, family= gaussian (link = log))

| **Coefficients** | **Estimate** | **Std. Error** | **t value** | **p value** |
| --- | --- | --- | --- | --- |
| Intercept | 2.90 | 0.25 | 11.65 | < 0.01 |
| Colony size | 0.01 | < 0.01 | 2.74 | 0.02 |
| Discovery | 0.01 | < 0.01 | 2.68 | 0.02 |
| Ant_antennae_impaired | 0.53 | 0.19 | 2.75 | 0.01 |

**Table S3: Comparison of relocation dynamics between antennae-impaired and control relocations.**

| **Parameters** | **Value** | **Statistics** |
| --- | --- | --- |
| Percentage of individual became explorers | Antennae-impaired: 4.74 ± 2.03  Control: 5.75 ± 4.57 | Mann-Whitney U test, U =43, N1 = 9, N2 = 10, p = 0.90 |
| Percentage of individual became leaders | Antennae-impaired: 11.96 ± 3.50  Control: 15.07 ± 4.27 | Mann-Whitney U test, U =60.5, N1 = 9, N2 = 10, p = 0.22 |
| Percentage of successful tandem runs | Antennae-impaired: 85.84 ± 11.40  Control: 80.14 ± 6.44 | Mann-Whitney U test, U=67.5, N1= 9, N2= 10, p = 0.07 |

**Table S4: Details of GLS analysis to compare the progression of leader recruitment over time in antennae impaired and control relocation**.

Model <- gls (Leader_recruitment ~ Category * Time, correlation = corAR1( form = ~ 1|colony), data= Leader_recruitment, method = “ML”)

| **Coefficients** | **Value** | **Std. Error** | **t value** | **p value** |
| --- | --- | --- | --- | --- |
| Intercept | 11.77 | 2.33 | 5.05 | < 0.01 |
| Antennae_impaired | 4.58 | 3.38 | 1.35 | 0.17 |
| Time | -0.03 | 0.04 | -0.08 | 0.39 |
| Antennae_impaired:Time | -0.08 | 0.05 | -1.52 | 0.12 |

**Table S5: Details of GLS analysis to compare the transport progression over time in antennae impaired and control relocation**.

Model <- gls (Transport ~ Category + Time, correlation = corAR1( form = ~ 1|colony), data= Transport, method = “ML”)

| **Coefficients** | **Value** | **Std. Error** | **t value** | **p value** |
| --- | --- | --- | --- | --- |
| Intercept | 7.13 | 1.98 | 3.59 | < 0.01 |
| Antennae_impaired | 2.51 | 2.88 | 0.87 | 0.38 |
| Time | 0.03 | 0.03 | 1.13 | 0.26 |
| Antennae_impaired:Time | -0.04 | 0.04 | -0.09 | 0.34 |

**Table S6: Details of GLM analysis to investigate the factors impacting the interruption occurred during individual tandem run.**

Model <- glm (Interruption ~ tandem-pair type, data= interruption, family= poisson)

| **Coefficients** | **Estimate** | **Standard error** | **z value** | **p value** |
| --- | --- | --- | --- | --- |
| Intercept | 0.57 | 0.15 | 3.75 | < 0.001 |
| Both-None (bn) | -2.76 | 0.59 | -4.63 | < 0.001 |
| Both-single(bs) | 60.83 | 0.32 | -2.64 | 0.008 |
| None-both (nb) | -0.61 | 0.18 | -3.39 | < 0.001 |
| None-none (nn) | -1.95 | 0.25 | -7.79 | < 0.001 |
| None-single (ns) | -1.27 | 0.20 | -6.22 | < 0.001 |
| Single-both (sb) | -0.32 | 0.19 | -1.69 | 0.09 |
| Single-none (sn) | -0.46 | 0.38 | -6.39 | < 0.001 |
| Single-single (ss) | -1.55 | 0.26 | -5.83 | < 0.001 |

**Pairwise comparison using “emmeans” package:**

| **Contrast** | **Estimate** | **Standard error** | **z ratio** | **p value** |
| --- | --- | --- | --- | --- |
| bb - bn | 2.763 | 0.597 | 4.630 | <.0001 |
| bb - bs | 0.834 | 0.316 | 2.641 | 0.0083 |
| bb - nb | 0.615 | 0.181 | 3.389 | 0.0007 |
| bb - nn | 1.952 | 0.250 | 7.792 | <.0001 |
| bb - ns | 1.268 | 0.204 | 6.217 | <.0001 |
| bb - sb | 0.322 | 0.190 | 1.690 | 0.0911 |
| bb - sn | 2.456 | 0.384 | 6.390 | <.0001 |
| bb - ss | 1.546 | 0.265 | 5.829 | <.0001 |
| bn - bs | -1.929 | 0.641 | -3.012 | 0.0026 |
| bn - nb | -2.147 | 0.586 | -3.664 | 0.0002 |
| bn - nn | -0.811 | 0.611 | -1.327 | 0.1844 |
| bn - ns | -1.495 | 0.593 | -2.519 | 0.0118 |
| bn - sb | -2.441 | 0.589 | -4.144 | <.0001 |
| bn - sn | -0.306 | 0.677 | -0.453 | 0.6509 |
| bn - ss | -1.216 | 0.617 | -1.971 | 0.0487 |
| bs - nb | -0.219 | 0.295 | -0.740 | 0.4591 |
| bs - nn | 1.118 | 0.342 | 3.270 | 0.0011 |
| bs - ns | 0.434 | 0.310 | 1.403 | 0.1606 |
| bs - sb | -0.512 | 0.301 | -1.702 | 0.0887 |
| bs - sn | 1.623 | 0.449 | 3.611 | 0.0003 |
| bs - ss | 0.713 | 0.353 | 2.019 | 0.0435 |
| nb - nn | 1.337 | 0.224 | 5.965 | <.0001 |
| nb - ns | 0.653 | 0.171 | 3.828 | 0.0001 |
| nb - sb | -0.293 | 0.154 | -1.905 | 0.0568 |
| nb - sn | 1.841 | 0.368 | 5.007 | <.0001 |
| nb - ss | 0.931 | 0.240 | 3.872 | 0.0001 |
| nn - ns | -0.684 | 0.243 | -2.818 | 0.0048 |
| nn - sb | -1.630 | 0.231 | -7.046 | <.0001 |
| nn - sn | 0.505 | 0.406 | 1.242 | 0.2142 |
| nn - ss | -0.405 | 0.296 | -1.370 | 0.1708 |
| ns - sb | -0.946 | 0.180 | -5.258 | <.0001 |
| ns - sn | 1.188 | 0.379 | 3.133 | 0.0017 |
| ns - ss | 0.278 | 0.258 | 1.079 | 0.2805 |
| sb - sn | 2.134 | 0.372 | 5.735 | <.0001 |
| sb - ss | 1.224 | 0.247 | 4.952 | <.0001 |
| sn - ss | -0.910 | 0.415 | -2.190 | 0.0285 |

**Table S7: Details of GLM analysis to investigate the factors impacting the time taken to complete individual tandem run.**

Model <- glm (Time ~ tandem-pair type, data= time, family= gaussian (link = log))

| **Coefficients** | **Estimate** | **Standard error** | **t value** | **p value** |
| --- | --- | --- | --- | --- |
| Intercept | 4.46 | 0.09 | 45.50 | < 0.001 |
| Both-None (bn) | -0.67 | 0.18 | -3.80 | < 0.001 |
| Both-single(bs) | -0.58 | 0.19 | -2.96 | 0.003 |
| None-both (nb) | -0.49 | 0.12 | -4.05 | < 0.001 |
| None-none (nn) | -1.01 | 0.15 | -6.71 | < 0.001 |
| None-single (ns) | -0.78 | 0.14 | -5.64 | < 0.001 |
| Single-both (sb) | -0.42 | 0.13 | -3.27 | 0.001 |
| Single-none (sn) | -1.13 | 0.20 | -5.634 | < 0.001 |
| Single-single (ss) | -0.90 | 0.18 | -5.120 | < 0.001 |

**Pairwise comparison using “emmeans” package:**

| **Contrast** | **Estimate** | **Standard error** | **t ratio** | **p value** |
| --- | --- | --- | --- | --- |
| bb - bn | 0.6708 | 0.176 | 3.805 | 0.0002 |
| bb - bs | 0.5804 | 0.196 | 2.958 | 0.0032 |
| bb - nb | 0.4912 | 0.121 | 4.050 | 0.0001 |
| bb - nn | 1.0115 | 0.151 | 6.714 | <.0001 |
| bb - ns | 0.7820 | 0.139 | 5.644 | <.0001 |
| bb - sb | 0.4173 | 0.128 | 3.270 | 0.0012 |
| bb - sn | 1.1284 | 0.200 | 5.634 | <.0001 |
| bb - ss | 0.9047 | 0.177 | 5.120 | <.0001 |
| bn - bs | -0.0905 | 0.224 | -0.403 | 0.6871 |
| bn - nb | -0.1796 | 0.163 | -1.101 | 0.2714 |
| bn - nn | 0.3406 | 0.186 | 1.831 | 0.0677 |
| bn - ns | 0.1112 | 0.176 | 0.630 | 0.5288 |
| bn - sb | -0.2535 | 0.168 | -1.510 | 0.1318 |
| bn - sn | 0.4576 | 0.228 | 2.006 | 0.0454 |
| bn - ss | 0.2338 | 0.208 | 1.126 | 0.2608 |
| bs - nb | -0.0892 | 0.184 | -0.483 | 0.6290 |
| bs - nn | 0.4311 | 0.205 | 2.103 | 0.0360 |
| bs - ns | 0.2017 | 0.196 | 1.028 | 0.3047 |
| bs - sb | -0.1630 | 0.189 | -0.864 | 0.3880 |
| bs - sn | 0.5481 | 0.244 | 2.248 | 0.0250 |
| bs - ss | 0.3243 | 0.225 | 1.443 | 0.1498 |
| nb - nn | 0.5203 | 0.135 | 3.854 | 0.0001 |
| nb - ns | 0.2908 | 0.121 | 2.396 | 0.0170 |
| nb - sb | -0.0739 | 0.109 | -0.679 | 0.4972 |
| nb - sn | 0.6372 | 0.189 | 3.375 | 0.0008 |
| nb - ss | 0.4135 | 0.164 | 2.528 | 0.0118 |
| nn - ns | -0.2295 | 0.151 | -1.522 | 0.1286 |
| nn - sb | -0.5942 | 0.141 | -4.221 | <.0001 |
| nn - sn | 0.1170 | 0.209 | 0.560 | 0.5758 |
| nn - ss | -0.1068 | 0.186 | -0.573 | 0.5669 |
| ns - sb | -0.3647 | 0.128 | -2.855 | 0.0045 |
| ns - sn | 0.3464 | 0.200 | 1.729 | 0.0844 |
| ns - ss | 0.1226 | 0.177 | 0.694 | 0.4881 |
| sb - sn | 0.7111 | 0.193 | 3.685 | 0.0003 |
| sb - ss | 0.4873 | 0.168 | 2.895 | 0.0040 |
| sn - ss | -0.2238 | 0.228 | -0.980 | 0.3277 |

**Table S8: Details of GLM analysis to investigate the factors impacting the tandem pair angular alignment during individual tandem run.**

Model <- glm (Angle ~ Followers’ antennae condition, data= angular alignment, family= gaussian (link = log))

| **Coefficients** | **Estimate** | **Std. Error** | **t value** | **p value** |
| --- | --- | --- | --- | --- |
| Intercept | 3.31 | 0.07 | 44.89 | < 0.001 |
| Follower_No-antennae impaired | -1.14 | 0.24 | -4.71 | < 0.001 |
| Follower_Single-antennae impaired | -0.55 | 0.14 | -3.81 | < 0.001 |

**Pairwise comparison using “emmeans” package:**

| **Contrast** | **Estimate** | **Standard error** | **t ratio** | **p value** |
| --- | --- | --- | --- | --- |
| Both vs None | 1.15 | 0.24 | 4.71 | < 0. 001 |
| Both vs Single | 0.55 | 0.14 | 3.81 | < 0. 001 |
| None vs Single | -0.59 | 0.26 | -2.27 | 0.03 |

**Table S9: Definition of the relocation dynamics parameters**.

| Parameters | Definitions |
| --- | --- |
| Discovery time | Time differences between the colony was placed inside the arena, the time at which 1st individual of the colony discovered the new nest. |
| Transportation time | Time differences between the 1st tandem pair and last tandem pair reached the new nest. |
| Successful tandem run | Tandem runs initiated from the old nest with a particular leader-follower pair and terminated at the new nest as the same pair. |

**SE: Mobility assay**

**Materials and Method**

Eleven *Diacamma indicum* colonies were collected from Nadia, West Bengal from April 2023 to April 2024 using the nest flooding method. Colonies were kept in a petri dish of 9 cm within the plastic box act as an artificial nesting site and were provided with *ad libitum* water and food consisting of ant cakes (Bhatkar & Whitcomb, 1970). Each colony of *D. indicum* consists of a reproductive individual called gamergate, who was distinguished by the presence of a pair of gemma at the second thoracic segment, several female workers and brood at different stages of development.

Experimental setup:

A 29 cm X 22 cm X 12 cm plastic box filled with white sand was used for all the mobility experiments. In the plastic box, a piece of paper with dimension 2 inches X 2 inches was kept and used as a reference scale for each video. The entire mobility assay experiment was recorded by a video recorder (Panasonic HC-V270). The aim of the experiment was to examine the impact of antennae restriction on the exploratory behaviour of the individual ants across colonies. Two sets of experiments were done to address this question.

Antennae impaired mobility assay:

Five colonies having 29.20 ± 10.42 adults were used to perform the experiment. Prior to conducting the experiment, individual ants from the colonies were received three types of treatments: Both antennae restricted (n = 19), left antennae restricted (n = 15) and right antennae restricted (n = 17). The process of antennal impairment was done 2 hours before the start of the experiment. The marked ants were collected in vials individually and given cold shock for 10 minutes by placing them in the ice bucket. This made the ants temporarily immobile which helped us in impaired the antennae. All the individuals experienced equal time of cold shock irrespective of being in both-antennae, left-antennae or right-antennae categories. A small drop of non-toxic enamel paint was applied at the base of the antenna of the ant to restrict antennal movement with a dissection pin. More specifically, the scape was attached to the head capsule with the paint. This treatment allowed the ants to move other segments or the flagellum of their antenna, but the reach of the antenna was compromised.

Control mobility assay:

Another six colonies with 62.16 ± 18.81 adults were used in control experiments. Four individuals were used from each colony for the experiment (n = 24). These individuals were neither exposed to cold shock nor antennae impairment. All the condition of the mobility assay remains identical across the treatment and control sets of experiment.

Behavioral observation:

Behavioral observation was conducted using video recorders. Each individual was placed in the sand box and three minutes video of exploration was recorded. The three minutes video was decoded using AnTracks (Version 1.018). To analyse the parameters at individual level, we extrapolated: total distance covered (pixels), and respective x and y coordinates (pixels) of the trajectories to measure the speed and farthest distance travelled by the individual ant respectively.

A digital image of the sand box was extracted from the video using AnTracks for every individual and used as a reference image for scaling in the ImageJ software (Verison 1.53), Using ImageJ, we extracted the speed and farthest distance explored in centimeters per second (cm/s) and centimeters respectively explored by the individual ant from each video. After tracking individual ant, the data was transferred to the Excel sheet for further analysis. To analyze the individual level parameters like speed, and farthest distance explored between antennae-impaired and control ants, a linear mixed model (LMM) and generalized linear mixed model (GLMM) was used respectively. Antennal condition was used as predictor to analyze the “speed (cm/s)” and “farthest distance explored (cm)” as the response variable and colony ID as a random variable. However, “squared-transformed farthest distance (cm)” as a response variable was used to better fit the model. Multcomp package (R Version 4.3.2) using Tukey method has been used for pairwise analysis.

**Results**

Mobility assay:

Exploration behavior of all the individual ants of control and treatment colonies were observed for 3 mins. After analyzing the parameter, we found that there is no significant difference between control (1.53 ± 0.633 cm/sec) and both impaired (1.08 ± 0.53 cm/sec) *(LMM, est = -0.37, p = 0.16*, see table SE1, fig. 1A*)* and single impaired ants *(LMM, est = 0.02, p = 0.93*, see table S1*).* However, pairwise comparison has given a significant difference between the speed of both (1.08 ± 0.53 cm/sec) and single antennae impaired ants (Tukey method, est = -0.39 p = 0.04, see table SE2, fig. 1D). We also found that the farthest distance explored by the control ants (18.26 ± 3.93 cm) was significantly higher as compared to the distance explored by the both antennae impaired ants (14.24 ± 5.61 cm) and statistically comparable with single antenna-impaired ants (16.24 ± 4.95 cm) (G*LMM, est = -107.47, p < 0.05*, see table SE3 and SE4, fig. 1E).


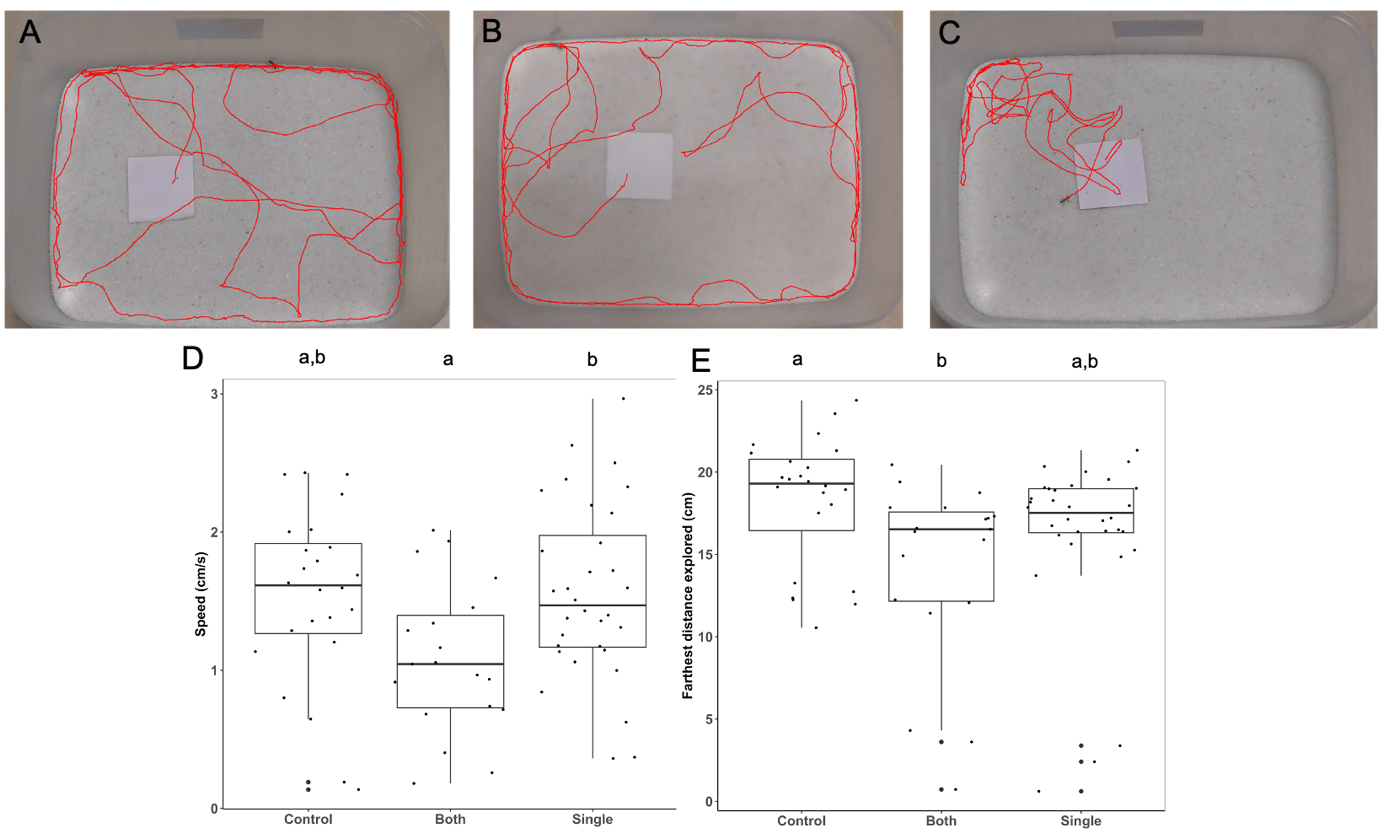


Figure 1. Mobility assay of individual ants. A) Representation of a trajectory of a control ant. B) Representation of a trajectory of single impaired antennae ant. C) Representation of a trajectory of both impaired antennae ant. D) Speed (cm/s) of control, both and single impaired ants. E) Farthest distance explored by control, both and single impaired ants. The black bold line denotes the median value, the box represents the interquartile range, and whiskers represent the 1.5 times the interquartile range. The jitters denote the data points. Significant differences between groups are represented by different alphabets placed above the boxes.

**Table SE1**: **Details of LMM analysis to investigate the impact of antennae impairment on the speed of individual exploring behaviour**

Model <- lmer (Speed ~ Type + (1 | Colony ID), data= Antennae_BS)

| **Coefficients** | **Estimate** | **Std. Error** | **t value** | **p value** |
| --- | --- | --- | --- | --- |
| **Control** | 1.53 | 12.34 | 9.04 | < 0.05 |
| **Both antennae impaired** | -0.37 | 12.78 | -1.46 | 0.16 |
| **Single antennae impaired** | 0.02 | 10.38 | 0.09 | 0.93 |

The response Speed denotes the speed of the individual ant in cm/s. The fixed effect is “Type” which consists three categories: control, both antennae impaired and single antennae impaired. The colony ID is used as random variable in the model.

**Table SE2**: **Details of pairwise analysis of LMM to investigate the impact of antennae impairment on speed of individual exploring behaviour**

| **Contrast** | **Estimate** | **Std. Error** | **z value** | **p value** |
| --- | --- | --- | --- | --- |
| **Both : Control** | - 0.37 | 0.25 | -1.46 | 0.30 |
| **Single : Control** | 0.02 | 0.24 | 0.09 | 0.99 |
| **Single : Both** | 0.39 | 0.16 | 2.47 | < 0.05 |

Pairwise comparison of speed of individual ants between three categories. control, both antennae impaired and single antennae impaired by tukeys’ method.

**Table SE3**: **Details of GLMM analysis to investigate the impact of antennae impairment on the farthest distance explored by individual ants**

Model <- glmer (Farthest Distance ~ Type + (1 | Colony ID), family = gaussian (link = identity) data= Antennae_BS)

| **Coefficients** | **Estimate** | **Std. Error** | **t value** | **p value** |
| --- | --- | --- | --- | --- |
| **Control** | 348.41 | 25.83 | 13.49 | < 0.05 |
| **Both antennae impaired** | -107.46 | 42.82 | -2.51 | < 0.05 |
| **Single antennae impaired** | -59.25 | 39.92 | -1.48 | 0.13 |

The response farthest distance denotes the farthest distance explored by the individual ant in cm. The fixed effect is “Type” which consist three categories: control, both antennae impaired and single antennae impaired. The colony ID is used as random variable in the model.

**Table SE4**: **Details of pairwise analysis of GLMM to investigate the impact of antennae impairment on the farthest distance explored by individual ants.**

| **Contrast** | **Estimate** | **Std. Error** | **z value** | **p value** |
| --- | --- | --- | --- | --- |
| **Both : Control** | -107.47 | 42.82 | -2.51 | < 0.05 |
| **Single : Control** | -59.25 | 39.39 | -1.48 | 0.29 |
| **Single : Both** | 48.22 | 30.18 | 1.59 | 0.24 |

Pairwise comparison of farthest distance explored by individuals between three categories. control, both antennae impaired and single antennae impaired by tukeys’ method.
